# Pembrolizumab-Associated Aortitis in a Patient With Triple-Negative Breast Cancer: A Case Report

**DOI:** 10.7759/cureus.103362

**Published:** 2026-02-10

**Authors:** Nicolas Ardila, Camila A Guerrero Bermúdez, Sergio A Tasco Múnera, Jessica Choi Park

**Affiliations:** 1 Internal Medicine, Universidad de Antioquia, Medellín, COL; 2 General Medicine, Universidad de La Sabana, Chía, COL; 3 Radiology, Universidad de Antioquia, Medellín, COL

**Keywords:** aortitis, immune checkpoint inhibitor, immune-related adverse events (irae), pembrolizumab, pembrolizumab side effect, triple-negative breast cancer (tnbc)

## Abstract

Immune checkpoint inhibitors have significantly improved outcomes across multiple malignancies; however, they can be associated with rare and potentially life-threatening immune-related adverse events. We report a case of a 47-year-old female with triple-negative breast cancer who developed acute epigastric and back pain with hemodynamic instability three days after receiving her fourth cycle of adjuvant pembrolizumab (last administered on July 14, 2025), leading to emergency department admission on July 17, 2025. Contrast-enhanced computed tomography revealed diffuse inflammatory thickening of the thoracic and abdominal aorta, consistent with aortitis, without aneurysm formation or stenosis. An extensive infectious and autoimmune workup was negative. Given the close temporal relationship with pembrolizumab exposure and exclusion of alternative etiologies, immune-mediated aortitis was suspected. High-dose intravenous corticosteroids followed by oral tapering led to rapid clinical improvement and normalization of inflammatory markers. Follow-up positron emission tomography-computed tomography demonstrated complete resolution of vascular inflammation, with no evidence of cancer recurrence. To our knowledge, this represents the first reported case of pembrolizumab-associated aortitis in a patient with triple-negative breast cancer, highlighting the importance of maintaining a high index of suspicion for immune-mediated vascular complications in patients receiving immune checkpoint inhibitors and underscoring the need for prompt diagnosis and treatment to prevent severe outcomes.

## Introduction

Immune checkpoint inhibitors (ICIs) have revolutionized the treatment of several solid and hematologic malignancies, leading to substantial improvements in survival across different cancer types [1]. These agents, which target the PD-1/PD-L1 or CTLA-4 pathways, are now used not only in metastatic settings but also in adjuvant and neoadjuvant therapies [2]. Pembrolizumab, a humanized IgG4 kappa monoclonal antibody that blocks the PD-1 receptor and activates T-cell-mediated antitumor responses, has shown significant clinical benefit in aggressive breast cancer subtypes, particularly triple-negative disease [3]. The KEYNOTE-355 and KEYNOTE-522 trials demonstrated improved survival and pathological complete response in PD-L1-positive tumors, leading to its approval in both early and advanced clinical settings [4,5].

Despite their efficacy, ICIs may lead to immune-related adverse events (irAEs) affecting nearly any organ system [6]. Vascular complications are uncommon, and large-vessel vasculitis, particularly aortitis, represents a rare but potentially life-threatening manifestation, given that its diagnosis is often challenging due to its low incidence and nonspecific clinical presentation, which may result in delayed recognition [7]. We report a case of pembrolizumab-associated aortitis in a patient with breast cancer, highlighting key diagnostic and management considerations.

## Case presentation

A 47-year-old premenopausal female was diagnosed with left-sided invasive ductal carcinoma of the breast, triple negative, cT3N1M0 (stage IIIB). Initial breast imaging revealed bilateral nodules, with a dominant lesion in the left breast measuring 46 mm. Germline BRCA1/2 testing was negative.

Neoadjuvant treatment was initiated on September 19, 2024, with pembrolizumab in combination with carboplatin and paclitaxel. After completing four cycles on November 24, 2024, treatment was continued with doxorubicin and cyclophosphamide. Due to local disease progression, the patient underwent left mastectomy with axillary lymph node dissection during this treatment course on January 10, 2025. Surgical pathology revealed a 20-mm residual tumor with negative margins and no nodal involvement (0/8 nodes).

Subsequently, adjuvant radiotherapy was administered between May 20, 2025, and June 10, 2025. During this period, pembrolizumab was reintroduced in the adjuvant setting, with the first cycle administered on May 10, 2025, and continued every three weeks thereafter. During the second cycle of this phase, the patient developed immune-related thyroiditis, which was managed conservatively. She received a total of four adjuvant pembrolizumab cycles, with the last infusion administered on July 14, 2025.

Three days after the most recent pembrolizumab administration, on July 17, 2025, the patient presented to the emergency department with acute epigastric pain radiating to the back, unrelieved by analgesics. She was hypotensive, unresponsive to fluid resuscitation, and required vasopressor support with norepinephrine, vasopressin, and dobutamine.

Initial laboratory evaluation revealed mild leukocytosis (13,200/mm³), normal liver and pancreatic function tests, elevated C-reactive protein (16.4 mg/dL), and metabolic acidosis (pH = 7.16, bicarbonate (HCO₃) = 16.4 mmol/L). Brain natriuretic peptide and troponin levels were within normal limits (Table 1).

**Table 1:** Laboratory findings. ALT: alanine aminotransferase; AST: aspartate aminotransferase; CRP: C-reactive protein; HCO3: bicarbonate; BNP: brain natriuretic peptide; ANAs: antinuclear antibodies; ANCAs: anti-neutrophil cytoplasmic antibodies; HIV: HIV antibodies; Hbs-Ag: hepatitis B virus surface antigen; HCV-Anti: hepatitis C virus antibodies; VDRL: venereal disease research laboratory.

Laboratory measurement	Value	Reference value
White blood cells	13,200/µL	4,500-11,000/µL
ALT	26 U/L	<25 U/L
AST	34 U/L	<40 U/L
Amylase	68 U/L	<120 U/L
Lipase	49 U/L	<160 U/L
CRP	16.4 mg/dl	<2 mg/dl
pH	7.16	7.35-7.45
HCO3	16.4 meq	22-26 meq
BNP	81 pg/ml	<100 pg/ml
Troponin	16.8 ng/L	<90 ng/L
ANAs	Negative	Negative
ANCAs	Negative	Negative
C3	98 mg/dl	90-180 mg/dl
C4	20.6 mg/dl	10-40 mg/dl
HIV	Negative	Negative
Hbs-Ag	Negative	Negative
HCV-Anti	Negative	Negative
VDRL	Negative	Negative

**Figure 1:**
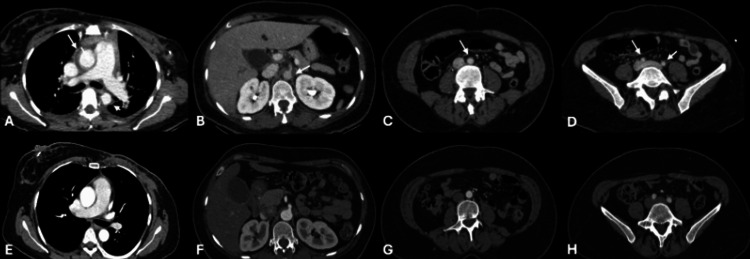
Contrast-enhanced computed tomography (venous phase) of the aortic axis before and after treatment. Upper row (A–D): Axial images from the pre-treatment study demonstrating laminar, concentric, hypodense, non-stenotic thickening of the aortic wall, consistent with periaortitis, involving the ascending and descending thoracic aorta (white arrows) (A), the suprarenal abdominal aorta with extension to the origin of the celiac trunk (B), the infrarenal abdominal aorta (C), and the common iliac arteries (D). Lower row (E–H): Axial images from the post-treatment study, obtained at the same anatomical levels, showing complete resolution of the wall thickening, with preserved aortic caliber and morphology, and no residual signs of vascular inflammation.

**Figure 2:**
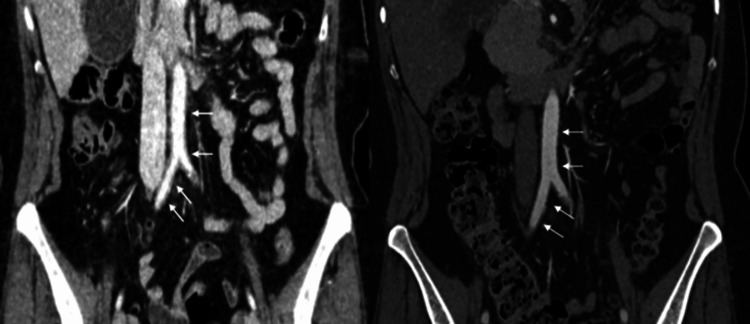
Contrast-enhanced computed tomography (venous phase) of the aortoiliac axis before (A) and after (B) treatment. Coronal image from the pre-treatment study (A) demonstrating laminar, hypodense, and non-stenotic thickening of the aortic wall, consistent with periaortitis, involving the infrarenal abdominal aorta and the common iliac arteries (white arrows). Coronal image from the post-treatment study (B), obtained at the same anatomical levels, showing complete resolution of the wall thickening (white arrows).

Pulmonary thromboembolism was initially suspected, and computed tomography angiography of the chest was performed. Although embolism was excluded, imaging findings were consistent with aortitis. The study was extended to the abdomen, demonstrating diffuse inflammatory involvement of the aortic arch, descending thoracic and abdominal aorta, as well as the visceral branches, without evidence of aneurysm or stenosis (Figures 1, 2).

Blood cultures were obtained, with *Staphylococcus saprophyticus* isolated in one of four bottles, which was interpreted as a contaminant. An extensive autoimmune panel, including antinuclear antibodies, antineutrophil cytoplasmic antibodies, and complement levels, as well as an infectious workup, including serologies for HIV, hepatitis B, hepatitis C, and syphilis, was negative (Table 1). Other potential drug-related causes of aortitis were also considered; notably, the patient did not receive granulocyte colony-stimulating factor (G-CSF) at any point during her oncologic treatment.

Given the absence of an infectious etiology and the close temporal relationship with pembrolizumab exposure, immune-mediated aortitis was suspected. Empiric broad-spectrum antibiotics (meropenem) were initiated and subsequently discontinued after negative cultures. High-dose intravenous methylprednisolone (500 mg daily for five days) was administered, followed by oral prednisolone (50 mg/day) with gradual tapering. The patient showed rapid clinical improvement, with resolution of abdominal pain, normalization of inflammatory markers, and progressive hemodynamic stabilization.

Pembrolizumab was permanently discontinued. After radiologic reassessment confirmed complete resolution of vascular inflammation and absence of cancer recurrence, adjuvant treatment was continued with capecitabine monotherapy. After 12 weeks of immunosuppressive therapy, while receiving oral prednisolone at a dose of 25 mg/day, follow-up positron emission tomography-computed tomography demonstrated complete resolution of the aortic inflammatory changes, with no residual metabolic activity (Figure 2).

## Discussion

ICIs are associated with irAEs in approximately half of treated patients, most of which are mild to moderate in severity [6]. Vasculitis, however, represents a rare but potentially life-threatening irAE, with an estimated incidence of 0.2% [7]. In the largest series published to date, small-vessel vasculitis predominate, including IgA vasculitis, cryoglobulinemic vasculitis, and ANCA-associated vasculitis, followed by large-vessel forms such as giant cell arteritis and aortitis, and, less frequently, medium-vessel entities, including cerebral vasculitis and polyarteritis nodosa. Notably, a considerable proportion of patients present with clinical and histopathological features of vasculitis that do not fully conform to traditional classification schemes [6].

It most often develops within the first months of therapy, typically between two and 18 months after ICI initiation, although delayed presentations up to 49 months have also been described [6,8]. Clinical presentations range from incidental findings of aortitis on routine oncologic imaging to nonspecific symptoms such as persistent fever, constitutional complaints, or elevated acute-phase reactants without an obvious cause. Patients may also present with chest, back, or abdominal pain, as in our case, or, in catastrophic scenarios, with aneurysm formation and rupture [9-11].

Both thoracic and abdominal segments may be involved, usually detected by fluorodeoxyglucose PET/CT or contrast-enhanced cross-sectional imaging rather than biopsy, which is technically challenging and not always feasible [6]. In this setting, the presence of vascular inflammation on imaging, together with temporal association with ICI initiation and exclusion of alternative causes, is generally sufficient to establish the diagnosis. In oncologic patients, the differential diagnosis must also include infectious and primary autoimmune etiologies [9], as well as paraneoplastic vasculitis [12], which may signal either active malignancy or recurrence. Furthermore, other drugs such as granulocyte colony-stimulating factor (e.g., pegfilgrastim) have been implicated in the development of transient aortitis and should be carefully considered [13].

Evidence regarding treatment is limited but relies primarily on high-dose glucocorticoids, which are considered first-line therapy and often lead to rapid clinical and radiological improvement [14,15]. However, corticosteroid use poses challenges in oncologic patients, as it may interfere with the antitumor efficacy of ICIs. Targeted therapies such as tocilizumab, an IL-6 receptor antagonist, have been used successfully as steroid-sparing agents, with reports of sustained remission and no apparent negative impact on cancer outcomes [6,8]. Balancing cancer control with adequate immunosuppression remains a key challenge, and multidisciplinary collaboration between oncologists, internists, and rheumatologists is essential to optimize outcomes.

To our knowledge, only a limited number of pembrolizumab-induced aortitis cases have been reported in the literature in patients with head and neck cancer, mesothelioma, and lung cancer [11,12,15-19]. The present report expands the spectrum of pembrolizumab-related large-vessel vasculitis by describing, for the first time, its occurrence in a patient with breast cancer. Although our patient showed complete clinical and radiological resolution after 12 weeks of immunosuppressive therapy, the relatively short duration of follow-up represents a limitation of this report. Given the risk of delayed vascular complications such as aneurysm formation or stenosis, long-term imaging surveillance is warranted even after apparent disease resolution. This case highlights the importance of maintaining a high index of suspicion for aortitis in patients receiving ICIs who develop systemic inflammation or vascular symptoms, and underscores the need for careful differential diagnosis, prompt initiation of immunosuppressive therapy, and structured long-term vascular follow-up to mitigate the risk of life-threatening complications.

## Conclusions

Pembrolizumab-associated aortitis is a rare but serious irAE that may present with nonspecific symptoms and hemodynamic instability. Early recognition, exclusion of alternative etiologies, and prompt initiation of immunosuppressive therapy are essential to prevent severe vascular complications. Clinicians should maintain a high index of suspicion for vascular toxicity in patients receiving immune checkpoint inhibitors. Multidisciplinary collaboration among oncology, internal medicine, rheumatology, and radiology teams is crucial for optimal management. In addition, given the risk of delayed vascular complications such as aneurysm formation or stenosis, long-term vascular follow-up should be considered even after apparent clinical resolution.
